# Comparison of dose calculated by an intensity modulated radiotherapy treatment planning system and an independent monitor unit verification program

**DOI:** 10.1120/jacmp.v4i3.2519

**Published:** 2003-06-01

**Authors:** J. J. Haslam, D. V. Bonta, A. E. Lujan, C. Rash, W. Jackson, J. C. Roeske

**Affiliations:** ^1^ Department of Radiation & Cellular Oncology University of Chicago Chicago Illinois 60637

**Keywords:** IMRT, monitor unit verification calculation

## Abstract

A comparison of isocenter dose calculated by a commercial intensity modulated radiation therapy treatment planning system and independent monitor unit verification calculation (MUVC) software was made. The percent disparity between the treatment plan and MUVC doses were calculated for 507 treatments (head and neck, prostate, abdomen, female pelvis, rectum and anus, and miscellaneous) from 303 patients. The MUVC calculated dose was, on average, 1.4% higher than the treatment planning dose, with a 1.2% standard deviation. The distribution of the disparities appeared to be Gaussian in shape with some variation by treatment site. Based on our analysis, disparities outside the range of ±3% about the mean value should be checked and resolved prior to treatment delivery.

PACS number(s): 87.53.–j, 87.66.–a

## INTRODUCTION

In radiation therapy, the monitor units (MUs) needed to deliver a treatment plan must be verified (often via a simple hand calculation) to correct potential errors prior to the start of treatment. This is especially true in intensity modulated radiation therapy (IMRT), where dose delivery can be quite complicated, with many beam angles and intricate intensity maps. The most comprehensive method of verifying the dose delivered to the patient is with a patient‐specific phantom study.^1^ In this analysis, the fluence maps from the patient plan are cast onto a phantom, and measurements (e.g., ion chamber and film dosimetry) are used to verify the calculated dose. However, this process is time consuming and may not be clinically feasible for every patient. In addition, the phantom geometry is often quite different from the patient geometry and some errors in the treatment planning process (e.g., incorrect definition of the patient external contour) may not be evident in a phantom measurement. Using a separate IMRT treatment planning system to perform independent dose verification has also been suggested.^2^ While this procedure would detect errors that might be missed in a phantom study, such an approach would be cost prohibitive and would increase the dosimetrist's work.

An alternative method involves the use of an independent dose calculation algorithm to perform a monitor unit verification calculation (MUVC). Such an MUVC has been recommended in the report of AAPM Task Group 40 on comprehensive quality assurance for conventional radiation oncology. As an extension of the previous recommendation, it has been proposed that a similar independent calculation of the dose to one point could be used as a quality assurance check for IMRT. At the University of Chicago, we use the commercially available RadCalc software (Lifeline Software, Inc., Tyler, TX) to verify the dose calculated by the CORVUS (NOMOS Corp., Sewickley, PA) treatment planning system. RadCalc uses a modified Clarkson integration technique to calculate the dose contribution by individual IMRT fields to the isocenter.^3^ The input required by the algorithm are the CORVUS treatment plan data (i.e., DMLC files, jaw settings, MU for each IMRT field, and depth to isocenter for each field). RadCalc uses independently measured beam data [Sc, Sp, TPR, ${\left(D/\text{MU}\right)}_{\text{ref}}$] and includes effects from multileaf collimator transmission and radiation field offset (difference in size between the light field and the radiation field due to transmission through the rounded ends of the multileaf collimator).

We have been using RadCalc as an independent MUVC for IMRT treatment planning for approximately two years, collecting a database of 303 patients with 507 treatment plans. In this paper we first compare dose calculated by the CORVUS planning system and RadCalc to ion chamber measurements. Next, we will analyze the variation in dose calculated by CORVUS and RadCalc for 507 treatments. Using these data, we will attempt to define a range of acceptable disparity between the treatment planning dose and the independent MUVC dose.

## MATERIALS AND METHODS

The RadCalc algorithm was originally developed at the University of Chicago by Kung, Chen and Kuchnir.^3^ In order to perform dose calculations for IMRT fields it introduces a modified Clarkson integration technique, which exploits the rotational symmetry of scattering in order to simplify the dose calculation. The RadCalc algorithm assumes that the external tissue contour of the patient is flat along a given beam direction. For dose calculations along the central axis, the IMRT fluence is replaced by an azimuthally averaged fluence. Thus, the Clarkson integration is carried out over annular sectors instead of pie sectors. In addition to the plan parameters provided by the CORVUS treatment plan, RadCalc requires the tissue depth to isocenter along the central axis at each beam angle. In our clinic, this distance is measured by the dosimetrist at the time that the digitally reconstructed radiographs (DRRs) are produced, using the AcQSim software (Philips Medical Systems, Cleveland, OH). The operator must manually contour the patient's skin, and choose the point which represents the treatment planning isocenter. RadCalc is capable of calculating dose at off‐axis points; however, in the majority of the cases in this study (99%), the calculation point was at the isocenter. RadCalc is also capable of performing heterogeneity corrections; however, none were used for the cases in this analysis.

For IMRT treatment planning, we use the commercially available CORVUS (Versions 3.0 and 4.0) planning system. To create a treatment plan the user is required to input the prescription dose, and specify the dose volume histograms for the target volume and critical structures. A simulated annealing algorithm is used to produce optimal intensity modulation profiles which are converted to MLC leaf positioning sequences as a function of monitor units.^4^ Doses are calculated in CORVUS using a finite pencil beam algorithm,^5^ with the treatment field divided into 1 cm×1 cm or 0.5 cm×0.5 cm beamlets. These are delivered using a Varian linear accelerator (Varian Associates, Palo Alto, CA) in step‐and‐shoot mode with an 80‐ or 120‐leaf collimator.

In order to validate the use of RadCalc in our clinic, we compared calculation and measurement for 35 phantom studies. Initially, a computed tomography (CT) scan was obtained of the Benchmark IMRT Phantom (Med‐Tec, Orange City, IA) with a $0.1\;{\text{cm}}^{3}$ ion chamber (PTW, Nuclear Associates, Hicksville, NY) in place. Next, the phantom was transferred to the CORVUS workstation and the fluence maps from individual patient plans were cast onto the phantom. We recorded both the dose to the isocenter (for comparison with RadCalc) and the average dose to the ion chamber as contoured on the CT scan (for comparison with measurement). The RTP files were then transferred from CORVUS to VARIS (Varian Associates, Palo Alto, CA) prior to plan delivery. All plans were delivered on a Varian CL 2100 C/D. The phantom was positioned on the treatment table such that the ion chamber was at isocenter, as defined by the lasers. Prior to each measurement, the ion chamber was calibrated to a standard dose resulting in a measurement uncertainty of approximately 1%.^6^

As of January 2003, a total of 303 patients with 507 separate treatment plans have been planned with CORVUS and verified using the RadCalc program. For each case, the dose disparity (the percent difference between the CORVUS and RadCalc dose) was calculated. The formula we used to calculate the dose disparity is shown in Eq. (1),

(1) $$\mathrm{Dose}\;\mathrm{disparity}=\frac{\mathrm{RadCalc}\;\mathrm{dose}-\mathrm{CORVUS}\;\mathrm{dose}}{\mathrm{CORVUS}\;\mathrm{dose}}\times 100.$$

The disparities of all 507 plans were then characterized by the mean and standard deviation of the distribution. The treatment plans were separated by site into the following categories: head and neck, prostate, abdomen, female pelvis, rectum and anus, and miscellaneous (including brain melanoma, spine, etc.) and the mean and standard deviation of the disparity in each site were determined. In selected sites, histograms were plotted and compared to a Gaussian distribution with the same mean and standard deviation. In order to determine if the distribution of disparities varied by treatment site, a two‐sample *t*‐test, with the assumption of unequal variances, was performed on pairs of groups to test whether the difference of the means was statistically significant. Additionally, a two‐sample *F*‐test was also performed on selected pairs to test whether the difference of the variances was statistically significant.

## RESULTS

**Figure 1:**
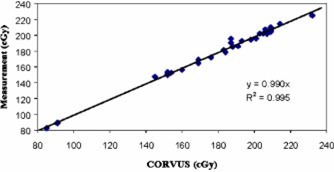
(Color) Plot of ion chamber measurement vs the calculated average dose to the contoured chamber.

**Figure 2:**
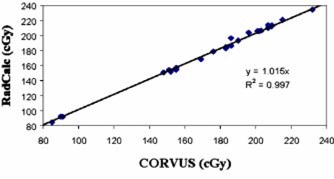
(Color) Plot of RadCalc MUVC vs the isocenter dose calculated by CORVUS.

In our validation of RadCalc, two comparisons were made. For measurements, we compared the CORVUS dose averaged over the volume of the ion chamber (as contoured) to the measurement (Fig. 1). Next, since RadCalc is a point calculation, we compared the RadCalc and CORVUS calculations at isocenter (Fig. 2). RadCalc and measurement were not directly compared as the former is a point calculation and the latter is averaged over a small volume. Both the ion chamber and RadCalc data show good agreement with CORVUS, as evidenced by the slopes of 0.990 and 1.015, respectively. Correlation coefficients are close to unity indicating the curve fit the data well. The ratios of measurement to calculated (CORVUS) dose values range from 0.97–1.04, while the ratios of RadCalc to CORVUS dose values range from 1.00–1.05.

**Figure 3:**
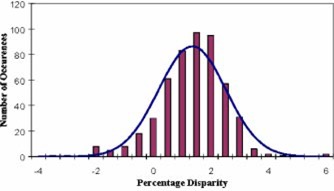
(Color) Histogram of disparities between RadCalc and CORVUS dose calculations, with fitted Gaussian. The mean value is 1.4% with a standard deviation of 1.2%.

**Figure 4:**
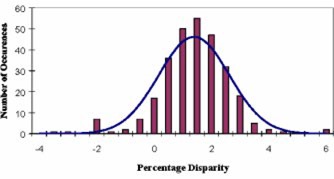
(Color) Histogram of disparities between CORVUS and RadCalc dose calculations for head and neck treatments, with fitted Gaussian. The mean value is 1.4% with a standard deviation of 1.2%.

**Figure 5:**
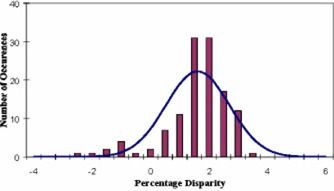
(Color) Histogram of disparities between CORVUS and RadCalc dose calculations for prostate treatments, overlaid with Gaussian fit. The mean value is 1.6% with a standard deviation of 1.1%.

In the analysis of 507 treatment plans, the mean disparity was calculated to be 1.4% with a standard deviation of 1.2%. Table I shows the mean disparity and standard deviation for the entire dataset (507 cases, all sites) as well as site‐specific results. Additionally, the 95% confidence intervals (CI) of the mean, and the range of disparities are listed. A histogram of the disparities for these 507 treatment plans, along with a plot of the Gaussian distribution that was fitted to the data are shown in Fig. 3. Histograms of the disparities for head and neck and prostate treatments are shown in Figs. 4 and 5, respectively. Histograms for the remaining sites are not presented because of their relatively small sample size.

**Table I:** Summary statistics of disparity between dose calculations using the CORVUS and RadCalc algorithms.

Site	Mean (%) ±Std. dev. (%)	95% CI of the mean (%)	Range (%)	# Cases
All	1.4±1.2	(1.3, 1.5)	(−3.3, 6.1)	507
Head and neck	1.4±1.2	(1.3, 1.5)	(−3.3, 6.1)	284
Prostate	1.6±1.1	(1.4, 1.8)	(−2.5, 3.3)	121
Abdomen	1.1 ±0.6	(0.9, 1.3)	(0, 2.4)	38
Miscellaneous	1.2±1.4	(0.7, 1.7)	(−1.7, 3.1)	28
Female pelvis	0.2±1.1	(−0.2, 0.8)	(−1.4, 2.4)	22
Rectum and anus	0.6±0.9	(0.1, 1.1)	(−0.9, 2.6)	14

Table I shows that head and neck, prostate, and miscellaneous cases all have similar means and standard deviations, as evidenced by the overlap of their 95% confidence intervals for the mean. The female pelvis and rectum and anus cases also have very similar means and standard deviations, as is shown by the overlap of their 95% confidence intervals. The miscellaneous group is composed of plans from many different treatment sites with very different field sizes and external patient contours. This nonuniformity makes it impossible to draw any meaningful conclusions from this group.

Based on the analysis above, we propose to divide the treatment sites into three groups: (1) head and neck and prostate, (2) female pelvis and rectum and anus, and (3) abdomen. Two tail *t*‐tests were performed for pairs of these groups, showing that the differences in the means (1 versus 2, 2 versus 3, and 1 versus 3) are statistically significant with *p*‐values <0.01. A two‐sampl *F*‐test shows that the difference in the variances between groups (1) and (3), and groups (2) and (3) are significant with *p*‐values <0.01. However, the difference in the variance between groups (1) and (2) is not significant, with a *p*‐value of 0.14.

We hypothesize that the female pelvis and rectum and anus groups have similar distributions with smaller mean disparity because both sites are in the pelvis where the treatment fields are large, and the patient surface is nearly flat over the width of the treatment field. We would expect the mean disparity and standard deviation of the head and neck group to be larger because of the rapidly varying external contours of this site. However, we expected the distribution of the prostate group to more closely resemble that of the female pelvis and rectum and anus because all these sites are in the pelvis. As we collect more data for these groups we will be able to determine if the difference is significant.

## DISCUSSION

The goal of this study was to compare the dose calculated by CORVUS with the dose calculated by RadCalc. In order to validate the use of RadCalc, ion chamber measurements and RadCalc doses were compared with CORVUS doses for 35 cases. The data show that the RadCalc algorithm tends to calculate a higher dose than the CORVUS treatment plan. In general, the RadCalc calculation is about 1.4% higher than the CORVUS point dose calculation. This can be attributed to the differences in the way CORVUS and RadCalc calculate dose. CORVUS makes full use of the 3D patient model to calculate dose to isocenter (and to all points, of course). RadCalc assumes that at each gantry angle, the patient is flat; that is, the patient surface is normal to the beam at each particular angle. If one approximates a patient outline as a circle or ellipse (at a given cross‐section), the RadCalc method will overestimate the amount of tissue at any given angle. This overestimate in tissue will result in an increase of the scatter contribution to the isocenter dose. Hence, RadCalc will tend to calculate a higher dose to isocenter relative to CORVUS. If we consider this mean disparity as the systematic error between the two dose calculation algorithms, we can shift the disparity distribution by subtracting the mean disparity from each value. The distribution of the disparities would then be approximated by a normal distribution with zero mean and the standard deviation, σ, calculated here.

Our results also suggest that the disparity may be site specific with head and neck and prostate, female pelvis and rectum and anus, and abdomen having significantly different mean values. Therefore, a site‐specific offset value may be necessary. However, given the relatively small number of patients in some of these sites, more data must be acquired before recommending implementation of a site‐specific offset.

The purpose of an independent monitor unit calculation is to identify treatment plans which could result in an incorrect dose being delivered to the patient. It is not intended to completely replace phantom measurements for quality assurance, and it should only be used after extensive commissioning and testing have been done to verify the reliability of the treatment planning dose calculation algorithm. However, it can be used to identify errors in the treatment planning process. For example, if the external surface (skin) of the patient were misidentified (i.e., due to the presence of a low density immobilization device) the depths in the treatment plan may be incorrect. Suppose, for example, the CT table were included as part of the patient's external contour. In a female pelvic case, this would add approximately 10 cm to the tissue depth in the posterior fields. Using the correct tissue depths, RadCalc would predict an isocenter dose that is 20% higher than the treatment planning system. Such an error would not have been detected by a phantom measurement. While the latest release of CORVUS (version 5.0) can output the source‐to‐surface distance (SSD), it is useful to independently verify these depths to identify potential errors in contouring. Additionally, some centers may use an independent system to generate the DRRs used to validate patient set‐up. In our own case, we use the AcQSim software to generate our DRRs. Use of this system requires placement of the isocenter to agree with that of CORVUS. Since we use our treatment depths obtained from AcQSim, RadCalc indirectly provides a method of verifying isocenter placement.

In comparing the dose disparity between CORVUS and RadCalc we would like to determine a disparity limit, above which all treatments should be reviewed. The report of AAPM Task Group 40 recommends that MUVC with a discrepancy of greater than 5% be checked and resolved before the treatment is delivered. Several published studies demonstrate that 5% is a reasonable limit. Watanabe^7^ reports on an analytical pencil beam kernel dose calculation algorithm for use in checking IMRT plans, for which calculated doses agree with planned doses within ±2% when the calculation point is in an uniform dose region. However, when the dose calculation point is in a high gradient region, the difference could be greater than 5%. Xing *et al.* report agreement in five cases between CORVUS planning doses and MUVC within 4%, with one case greater than 7%.^8^ Similar agreement has been shown between treatment planning doses, and phantom dose measurements. Ting and Davis^9^ report that most doses measured in an anthropomorphic phantom generally agreed within ±5% of plan doses, with some values near ±10%. It was considered acceptable if the absolute point dose measurement was within 5% of the plan dose. In a study of 92 patients, using phantom measurements, Tsai *et al.* also report that discrepancies of less than 5% were acceptable.^10^

From the results reported in this paper and elsewhere we suggest an acceptable discrepancy between CORVUS and RadCalc of ±3% about the mean value. Based on our analysis, this corresponds to 2.5σ, indicating that approximately 1.2% of the cases would require a more detailed analysis. Based on our experience, if the disparity between the treatment planning dose and the RadCalc dose is outside this limit, the physicist should first check whether the calculation point is in a high dose gradient region and if the treatment depths are correct. If the calculation point is in a high dose gradient region, then a point in a low dose gradient region (i.e., target) should be selected, and an additional MUVC should be performed. Likewise, if the depths are incorrect, the external contour should be investigated for inclusion of immobilization devices, treatment table, etc. Additionally, in such cases, phantom measurements should also be performed.
